# Reconfigurable Multifunctional Metasurface Hybridized with Vanadium Dioxide at Terahertz Frequencies

**DOI:** 10.3390/ma11102040

**Published:** 2018-10-19

**Authors:** Ling Wang, Weijun Hong, Li Deng, Shufang Li, Chen Zhang, Jianfeng Zhu, Hongjun Wang

**Affiliations:** 1 Beijing Laboratory of Advanced Information Networks, Beijing University of Posts and Telecommunications, P.O. Box. 171, Beijing 100876, China; lingwang@bupt.edu.cn (L.W.); lisf@bupt.edu.cn (S.L.); zhangchenzc@bupt.edu.cn (C.Z.); zhujianfeng@bupt.edu.cn (J.Z.); wanghongjun@bupt.edu.cn (H.W.); 2 Beijing Key Laboratory of Network System Architecture and Convergence Acknowledgment, Beijing University of Posts and Telecommunications, P.O. Box. 171, Beijing 100876, China

**Keywords:** reconfigurable metasurface, multifunction, vanadium dioxide, terahertz frequency

## Abstract

Driven by the continuous demand for system integration and device miniaturization, integrating multiple diversified functions into a single metasurface hybridized with the tunable metaparticle is highly demanding at terahertz (THz) range. However, up to now, because of the limitation of the tunable metaparticle at terahertz range, most of the metasurfaces feature a single function only or process similar functionalities at a single frequency. A reconfigurable multifunctional metasurface which can realize the switch of transmission and reflection and manipulate the linearized polarization state of electromagnetic waves simultaneously over a controllable terahertz frequency range based on the vanadium dioxide was designed for the first time in the paper. The numerical result demonstrates the validity of the appropriately designed metasurface. Simulation results show that the reconfigurable and multifunctional performance of this metasurface can be acquired over 1.59 THz to 1.74 THz without re-optimizing or re-fabricating structures, which effectively extends the operating frequencies. The proposed metasurface holds potential for electromagnetic wave manipulation and this study can motivate the realization of the wideband multifunctional metasurface and the software-driven reconfigurable metasurface at terahertz frequencies.

## 1. Introduction

As the two-dimensional (2D) version of metamaterials [1,2,3,4,5], metasurface [6,7,8,9] has been overwhelmingly investigated. Recently, driven by the continuous demand for system integration and device miniaturization, integrating multiple diversified functions into a single metasurface is highly demanding at terahertz (THz) range. However, up to now, most of the reported metasurface feature a single function only or process similar functionalities at a single terahertz frequency because of the limit of tunable metaparticles [10,11,12,13,14,15,16,17,18,19]. In this regard, the metasurface integrating different functions over a multiwavelength or wide wavelength range based on tunable metaparticles has become an emerging research area that requires dealing with formidable challenges at terahertz frequencies.

Previous research was mainly focused on the multifunctional metasurface without tunable metaparticles; however, the multifunctionality can only be acquired at different polarization states, incident angles or wavelengths [20,21,22,23,24,25]. Recent efforts have started to be devoted to the design of the reconfigurable multifunctional metasurface by hybridizing metasurfaces with tunable metaparticles, such as the vanadium dioxide (VO_2_), graphene, liquid crystal, micro-electromechanical system (MEMS), varactor diode and PIN diode [26,27,28,29,30,31,32,33,34,35]. It is widely known that manipulation of the polarization state of electromagnetic (EM) waves [36,37,38,39,40,41] and the switch of the amplitude of transmission and reflection [42,43,44,45] are of paramount importance in both THz scientific and engineering applications. Thus, it makes a great significance to integrate these two functions into a single metasurface. Although a metasurface with the PIN diode has been proposed to achieve these two functions at a fixed single microwave frequency [46] in the THz region, existing metasurfaces cannot realize both of these functions simultaneously because of the limit of tunable metaparticles. To realize the switch of transmission and reflection, the metaparticle must exhibit an insulator-to-metal transition. The MEMS, varactor diode and PIN diode are used in the microwave region. Although the graphene and liquid crystal can be used in the THz region, the graphene is of great loss compared to VO_2_ and the liquid crystal cannot achieve the perfect insulator-to-metal transition in the THz region [47,48,49,50].

Therefore, we design a reconfigurable metasurface working at tunable THz frequencies with diversified functionalities based on VO_2_, which can exhibit an insulator-to-metal transition around 67 degrees driven by thermal effect [26,51,52,53,54], electrical tuning [55,56] or optical tuning [57]. Generally, the VO_2_-based metasurface can be fabricated by photolithography and the resistive heater or the external CW (continuous-wave) laser can be used to control the temperature of VO_2_. The unit cell of the proposed metasurface consists of simple three layers, which from top to bottom are the gold resonator hybridized with VO_2_, a dielectric layer and a gold substrate also embedded with VO_2_. The metallic wire at the top layer is related to the polarization of waves and the hybridized VO_2_ is used to dynamically tune the resonant frequency. VO_2_ at the bottom layer is used to switch the state of waves between transmission and reflection. In order to understand the contributions of each VO_2_ and demonstrate the performance of the proposed metasurface, three other types of unit cells are designed. The EM interference model [58,59] is used to illustrate the underlying mechanism of the proposed metasurface and the analytical technique is based on tracking the various Fabry-Perot-like scattering processes within the structure. Simulation results demonstrate that the appropriately designed metasurface can manipulate the linearized polarization state of EM waves and simultaneously realize the switch of transmission and reflection by utilizing the insulator-to-metal transition in VO_2_ inserted at the gap of bottom metallic gratings. The operating frequency can be dynamically tuned from 1.59 THz to 1.74 THz without re-optimizing or re-fabricating structures of resonators, while the conductivity of the VO_2_ loaded on the top metal resonator varies between 2 × 10^5^ S/m and 2 × 10^2^ S/m. The high performance of diversified functionalities with transmission-reflection switching and polarization control can be maintained across 1.59–1.62 THz and 1.71–1.74 THz. Thus, the dynamical control of the working frequency effectively extends operating frequencies of this metasurface.

A reconfigurable multifunctional metasurface hybridized with vanadium dioxide at terahertz frequencies is proposed for the first time in this paper. This metasurface can realize the switch of transmission and reflection and manipulate the linearized polarization state of electromagnetic waves simultaneously over a controllable terahertz frequency range without re-optimizing or re-fabricating structures. The proposed metasurface holds great potential for EM wave manipulation and can motivate the realization of the wideband multifunctional metasurface and the software-driven reconfigurable metasurface which have huge fascinations and prospects to conveniently realize complex system integration and device miniaturization with low costs at THz frequencies. This study can pave the way to many practical applications such as telecommunications, sensing and diagnostics, nanoelectronics, antennas and automotive.

## 2. The Design of Reconfigurable Multifunctional Metasurface

**Figure 1:**
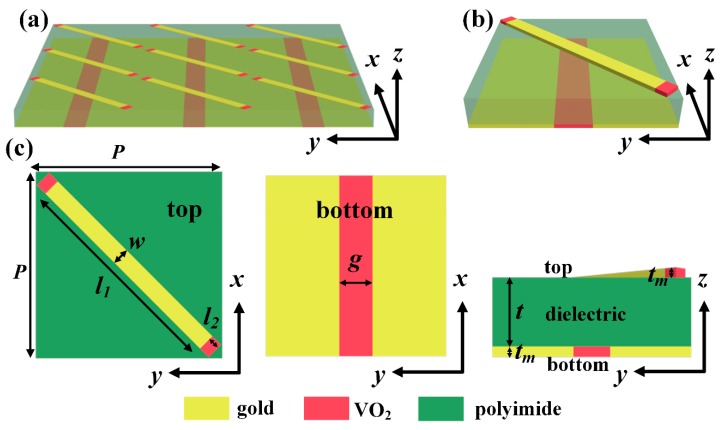
(**a**) Schematic view of the designed frequency-tunable reconfigurable multifunctional metasurface; (**b**) The unit cell of the metasurface composed of three layers; (**c**) Geometric parameters are the following: *P* = 54 μm, *l*_1_ = 70 μm, *l*_2_ = 3.5 μm, *w* = 5 μm, *g* = 10 μm, *t* = 9 μm, *t_m_* = 0.2 μm.

The unit cell of the proposed reconfigurable multifunctional metasurface is sketched in Figure 1. The unit cell consists of three simple layers, which, from top to bottom, are the gold resonator hybridized with VO_2_, a dielectric layer and a gold substrate, also embedded with VO_2_. The metallic wire at the top layer is related to the polarization of waves and the hybridized VO_2_ is used to dynamically tune the resonant frequency. VO_2_ at the bottom layer was used to switch the state of waves between transmission and reflection. The resonator was rotated 45° counterclockwise along the positive x-axis. The unit cell was periodically arranged in both the x-direction and y-direction. 

The period of the unit cell is *P* = 54 μm. The dielectric layer is made up of a lossy polyimide which was assumed to have the relative permittivity of 2.4 + 0.005*i* with a thickness of *t* = 8 μm. The gold was taken as a lossy metal with a conductivity of σ_Au_ = 4.561 × 10^7^ S/m and is 0.2 μm thick. Generally, the complex dielectric property of VO_2_ in the THz range can be described by the Bruggeman effective-medium theory (EMT). For simplicity, VO_2_ can be modeled with the Drude model with frequency-independent conductivity. The relative permittivity of VO_2_ is about nine in the insulating state, while the conductivity is smaller than 200 S/m and as high as an order of 10^5^ S/m in the metallic state [26,51,52,53,54]. The thickness of VO_2_ is identical to that of gold.

**Figure 2:**
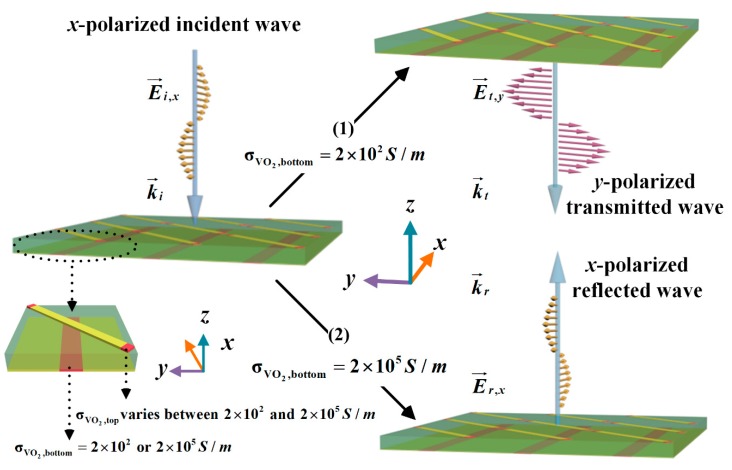
The illustration of the designed metasurface and expected multiple electromagnetic functionalities. The conductivity of VO_2_ on the top layer varies between 2 × 10^2^ S/m and 2 × 10^5^ S/m. By varying the $\sigma_{{\mathrm{VO}}_{2},\mathrm{top}}$, the resonant frequency is expected to be dynamically tuned. (1) When $\sigma_{{\mathrm{VO}}_{2},\mathrm{bottom}}$ = 2 × 10^2^ S/m, the incident THz wave is rotated by 90° to its orthogonal polarization state after transmitting through the metasurface. (2) When $\sigma_{{\mathrm{VO}}_{2},\mathrm{bottom}}$ = 2 × 10^5^ S/m, the linearly polarized incident wave will be reflected by the metasurface with the same linear polarization.

Figure 2 shows the illustration of the designed metasurface and expected multiple electromagnetic functionalities. By independently controlling the conductivity of the top and bottom VO_2_, the metasurface was expected to manipulate the state of incident waves between transmission and reflection, the polarization of the reflected or transmitted waves and dynamically tune the resonant frequency. As shown in Figure 2, a linearly polarized terahertz plane wave normally illuminates on the metasurface along the negative direction of the *z*-axis. (1) When the VO_2_ inserted at the gap of bottom metallic gratings was in the insulating state with $\sigma_{{\mathrm{VO}}_{2},\mathrm{bottom}}$ = 2 × 10^2^ S/m, the incident THz wave was rotated by 90° to its orthogonal polarization state after transmitting through the metasurface. (2) When the bottom VO_2_ was in its fully metallic state with $\sigma_{{\mathrm{VO}}_{2},\mathrm{bottom}}$ = 2 × 10^5^ S/m, the linearly polarized incident wave would be reflected by the metasurface with the same linear polarization. Simultaneously, by varying the conductivity of VO_2_, which is involved into each metallic resonator on the top of this metasurface between $\sigma_{{\mathrm{VO}}_{2},\mathrm{top}}$ = 2 × 10^2^ S/m and 2 × 10^5^ S/m, the resonant frequency could be dynamically tuned and thus the working frequency range can be extended. The performance of the proposed metasurface will be discussed and demonstrated in Section 4.

**Figure 3:**
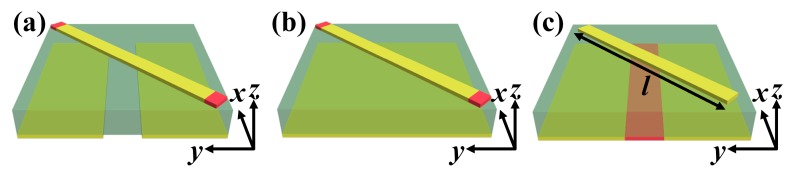
Perspective view of unit cells for demonstrating the performance of the proposed metasurface. (**a**) Metallic gratings without VO_2_; (**b**) Metallic substrate; (**c**) Metallic resonator without VO_2_, *l* = 70 μm or 77 μm.

In order to understand the contribution of each VO_2_ and demonstrate the performance of the proposed metasurface, we used air and gold to characterize the insulating state and all-metallic state of VO_2_, respectively, in the simulation. Three other types of unit cells were designed, as shown in Figure 3. Dimensions of these unit cells were the same as that in Figure 1, except the length *l* in Figure 3c. One could observe that in contrast to the unit cell in Figure 1, the bottom metallic grating with VO_2_ was replaced with the metallic grating without VO_2_ and metallic substrate, respectively, in Figure 3a,b; the VO_2_ at the top layer is removed in Figure 3c.

Based on the above description, it should be clear that VO_2_ at the bottom layer was used to switch the state of waves between transmission and reflection, and VO_2_ at the top layer was used to dynamically tune the resonant frequency. Therefore, the contribution of VO_2_ at the bottom layer can be understood by setting the conductivity of VO_2_ at the top layer to a certain value, while VO_2_ at the bottom layer was in the insulating state or its all-metallic state, respectively. Simultaneously, by contrasting the performance of the unit cell in Figure 1 with VO_2_ at the bottom layer in the insulating state, or its all-metallic state to unit cells in Figure 3a,b, respectively, the performance of transmission-reflection switching of the proposed metasurface could be verified. The contribution of VO_2_ at the top layer can be understood by comparing the operating frequency of the designed metasurface when the conductivity of VO_2_ at the top layer is in its insulating or metallic state, respectively. By contrasting the performance of the unit cell in Figure 1 to the unit cell in Figure 3c with *l* = 70 μm or 77 μm, the operating frequency tunability of the proposed metasurface could be verified.

## 3. Mechanism of the Reconfigurable Multifunctional Metasurface

**Figure 4:**
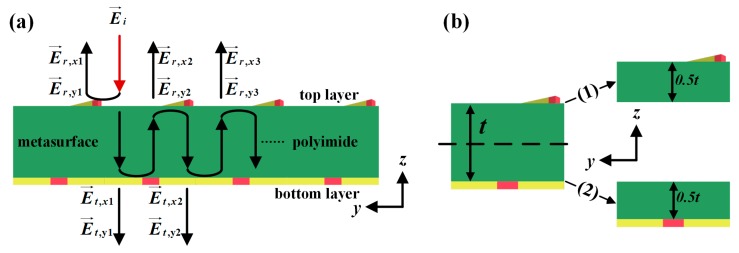
(**a**) Schematic of the EM interference model. (**b**) Decomposition of the unit cell: (1) The top layer with a 0.5*t*-thick lossy polyimide substrate; (2) The bottom layer with a 0.5*t*-thick lossy polyimide substrate.

Compared with many complex unit cells of reconfigurable multifunctional metasurfaces, the unit cell of the proposed metasurface is maybe the simplest and most effective structure which can greatly reduce the manufacturing complexity and effectively acquire multifunctionalities. The EM interference model [58,59] was used to illustrate the underlying mechanism of the three-layered reconfigurable multifunctional metasurface and the analytical technique is based on tracking the various Fabry-Perot-like scattering processes within the structures. From Figure 4a, we can see that, when the incident wave ${\vec{E}}_{i}$ illuminates onto the top layer, a portion is reflected by the top layer and is converted to an y-polarized wave or remain an x-polarized wave, while the other portion can propagate through the top layer and illuminates onto the bottom layer. Then a portion of these waves can penetrate the bottom layer with the x-polarized and the y-polarized wave, whereas the other portion is reflected by the bottom layer. A portion of these reflected waves will propagate through the top layer with x-polarized and y-polarized components, and the other portion will be reflected by the top layer and go back to interact with the bottom layer again. It is clear that this process is in an infinite loop.

To verify the working mechanism of the proposed reconfigurable multifunctional terahertz metasurface, as shown in Figure 4b, the unit cell of this metasurface is decomposed into two components: (1) the top layer with a 0.5*t*-thick lossy polyimide substrate; (2) the bottom layer with a 0.5*t*-thick lossy polyimide substrate. Dimensional parameters of this unit were same as those in Figure 1c. Their respective electromagnetic properties and the interaction in between were investigated based on the simulated scattering parameters. In the simulation, periodic boundary conditions were set in the x-direction and y-direction and were open in the z-direction under the condition of free space and the x-polarized and y-polarized incident wave was applied in the unit cell. Their respective 4 × 4 scattering matrices were as follows:

(1) $$S=\left(\begin{array}{cccc} {\vec{t}}_{xx} & {\vec{t}}_{xy} & {\overset{\leftarrow }{r}}_{xx} & {\overset{\leftarrow }{r}}_{xy}\\ {\vec{t}}_{yx} & {\vec{t}}_{yy} & {\overset{\leftarrow }{r}}_{yx} & {\overset{\leftarrow }{r}}_{yy}\\ {\vec{r}}_{xx} & {\vec{r}}_{xy} & {\overset{\leftarrow }{t}}_{xx} & {\overset{\leftarrow }{t}}_{xy}\\ {\vec{r}}_{yx} & {\vec{r}}_{yy} & {\overset{\leftarrow }{t}}_{yx} & {\overset{\leftarrow }{t}}_{yy} \end{array}\right)\;$$

where subscripts of transmission coefficients *t_ji_* and reflection coefficients *r_ji_* indicate the incident polarization *i* and the transmitted or reflected polarization *j*. The complex scattering coefficients carry the phase information about the top layer, bottom layer and the polyimide substrate. The arrows atop indicates the incidents propagation along the $-z\;(\vec{•})$ or $+z\;(\overset{\leftarrow }{•})$ direction. It is worth noting that, when the unit cell of this metasurface decomposed into two components, the coupling between the top and bottom layer was not considered, which will cause some error between the simulation and calculation results. Increasing the thickness *t* of the polyimide substrate would weaken the coupling effect between the top and bottom layer and the error would also be smaller.

It is clear that the process described above is an infinite loop. In addition, a 4 × 4 scattering matrices of two components in Figure 4b can be obtained by simulation. According to the simulation result, we can observe that all of the scattering coefficients used to calculate the overall ${\vec{T}}_{yx}^{\left(1)(2\right)}$ and ${\vec{R}}_{xx}^{\left(1)(2\right)}$ cannot be ignored. Thus, if the overall ${\vec{T}}_{yx}^{\left(1)(2\right)}$ and ${\vec{R}}_{xx}^{\left(1)(2\right)}$ are approximated by particular equations, there will be great errors.

**Figure 5:**
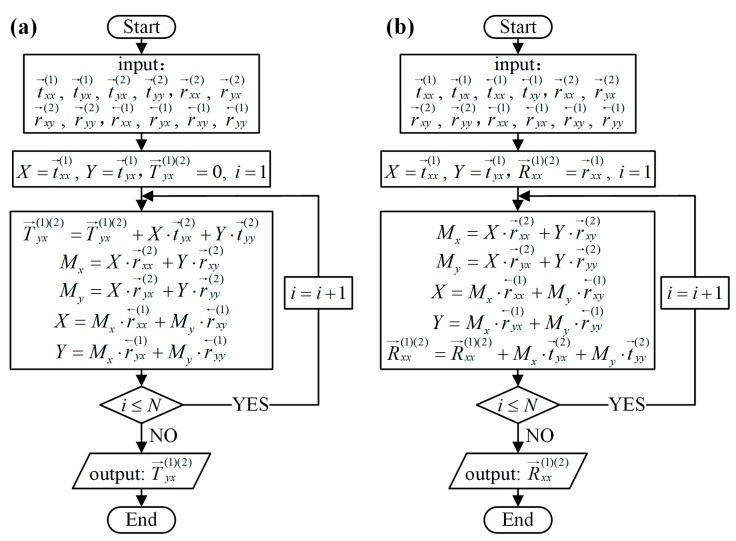
MATLAB flowcharts. (**a**) The flowchart for the analytical calculation of ${\vec{T}}_{yx}^{\left(1)(2\right)}$; (**b**) The flowchart for the analytical calculation of ${\vec{R}}_{xx}^{\left(1)(2\right)}$.

Therefore, for the most accurate calculation of the overall ${\vec{T}}_{yx}^{\left(1)(2\right)}$ and ${\vec{R}}_{xx}^{\left(1)(2\right)}$, the flowcharts in Figure 5 were used to analogy this infinite loop process mentioned above. The overall ${\vec{T}}_{yx}^{\left(1)(2\right)}$ and ${\vec{R}}_{xx}^{\left(1)(2\right)}$ can be calculated by MATLAB based on flowcharts instead of approximate formulas. As Figure 5 shows, firstly, scattering coefficients used to calculate ${\vec{T}}_{yx}^{\left(1)(2\right)}$ or ${\vec{R}}_{xx}^{\left(1)(2\right)}$ were inputted. Then the *N* iterations were used to analogy the infinite loop process. At last, the overall ${\vec{T}}_{yx}^{\left(1)(2\right)}$ and ${\vec{R}}_{xx}^{\left(1)(2\right)}$ can be output. The bigger the value of *N*, the more accurate the calculated result.

## 4. Results and Discussion

In the simulation, periodic boundary conditions were set in the x and y directions and were open in the *z*-direction under the condition of free space. The Floquet excitation port was applied in the unit cells, based on the commercial software ANSYS HFSS (2016.1, ANSYS, Canonsburg, PA, USA). The unit cell was normally illuminated by an x-polarized incident light, as illustrated in Figure 2. First, the property of transmission-reflection switching and polarization control of this multifunctional metasurface was examined by simulating the unit cells mentioned in Figure 1 and Figure 3a,b. The conductivity of VO_2_ at the top layer was assumed as 2 × 10^5^ S/m and the VO_2_ inserted at the gap of the bottom metallic gratings was in the insulating state and its all-metallic state 2 × 10^2^ S/m and 2 × 10^5^ S/m, respectively.

**Figure 6:**
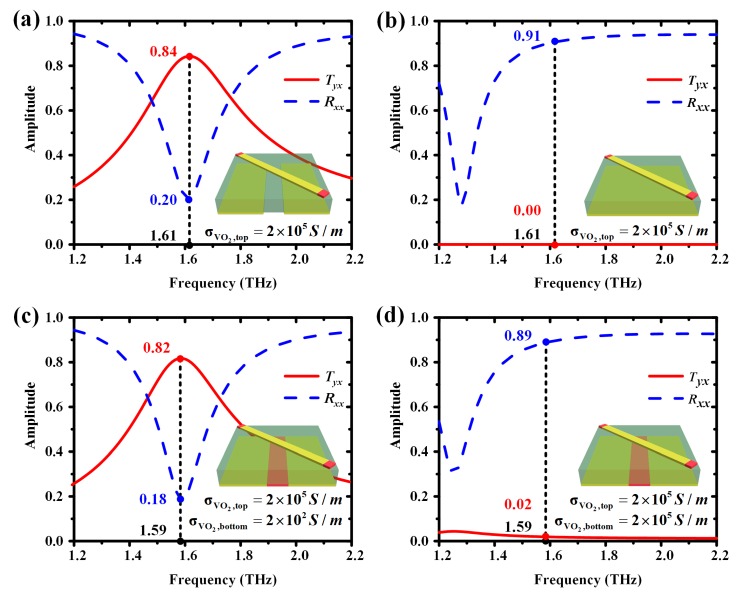
The simulated amplitude of the transmitted coefficient *T_yx_* (red-solid line) and the reflected coefficient *R_xx_* (blue-dashed line) of unit cells. All of the conductivity of VO_2_ at the top layer was assumed as $\sigma_{{\mathrm{VO}}_{2},\mathrm{top}}$ = 2 × 10^5^ S/m. (**a**) Metallic gratings without VO_2_; (**b**) Metallic substrate; (**c**) The unit cell of designed multifunctional terahertz metasurface. The VO_2_ at the bottom layer is in the insulating state with $\sigma_{{\mathrm{VO}}_{2},\mathrm{bottom}}$ = 2 × 10^2^ S/m; (**d**) The unit cell of designed multifunctional terahertz metasurface. The VO_2_ at the bottom layer is in its all-metallic state with $\sigma_{{\mathrm{VO}}_{2},\mathrm{bottom}}$ = 2 × 10^5^ S/m.

Figure 6 presents the amplitude of the transmitted coefficient *T_yx_* and the reflected coefficient *R_xx_* of these three unit cells. As seen from Figure 6a,b, the highest amplitude of *T_yx_* was 0.84, while the amplitude of *R_xx_* was 0.2 at 1.61 THz with a bottom metallic grating layer. The amplitude of *T_yx_* was 0, while the amplitude of *R_xx_* was 0.91 at 1.61 THz with a metallic substrate. As shown in Figure 6c,d at the resonant frequency 1.59 THz, when VO_2_ at the bottom layer was in the insulating state with $\sigma_{{\mathrm{VO}}_{2},\mathrm{bottom}}$ = 2 × 10^2^ S/m, the amplitude of *T_yx_* was 0.82 while the amplitude of *R_xx_* was 0.18. When VO_2_ at the bottom layer was in the all-metallic state with $\sigma_{{\mathrm{VO}}_{2},\mathrm{bottom}}$ = 2 × 10^5^ S/m, the amplitude of *T_yx_* was 0.02 while the amplitude of *R_xx_* was 0.89. Thus, it clearly shows that the performance of the VO_2_ at the bottom layer with $\sigma_{{\mathrm{VO}}_{2},\mathrm{bottom}}$ = 2 × 10^2^ S/m and 2 × 10^5^ S/m was almost the same as the ideal insulating and metallic state.

Therefore, the proposed multifunctional terahertz metasurface cannot only switch the state of waves between transmission and reflection, but also change the polarization of EM waves with high efficiency at the designed frequency.

Then, the operating frequency tunability of the proposed metasurface was investigated by simulating unit cells mentioned in Figure 1 and Figure 3c. The bottom VO_2_ underwent the insulator-to-metal transition. The conductivity of the top VO_2_ in Figure 1 varied between 2 × 10^2^ S/m and 2 × 10^5^ S/m. The length of the gold resonator in Figure 3c is 70 μm and 77 μm, respectively.

**Figure 7:**
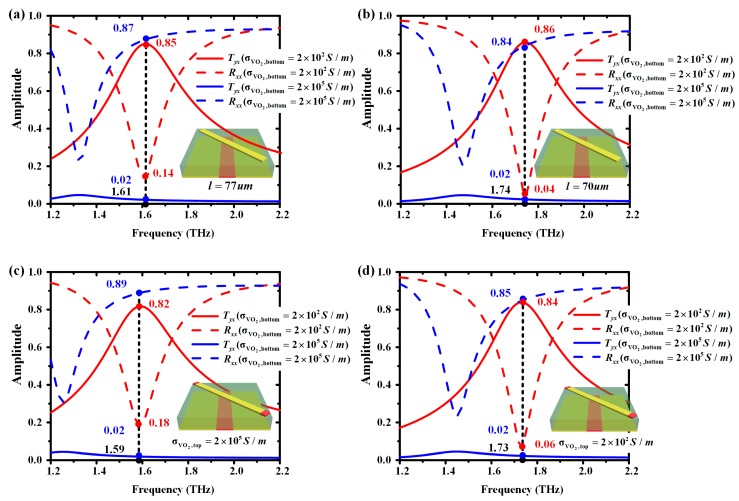
The simulated amplitude of the transmitted coefficient *T_yx_* (solid line) and the reflected coefficient *R_xx_* (dashed line) of unit cells. (**a**) *l* = 77 μm; (**b**) *l* = 70 μm; (**c**) σ_VO2,top_ = 2 × 10^5^ S/m; (**d**) σ_VO2,top_ = 2 × 10^2^ S/m.

The simulated *T_yx_* and *R_xx_* are plotted in Figure 7. It can be found from Figure 7a,b that as the *l* decreased from 77 μm to 70 μm, the working frequency increased from 1.61 THz to 1.74 THz. Simultaneously, the transmissivity of cross-polarization and reflectivity of co-polarization were greater than 80% or less than 15% in the operating frequency. Figure 7c,d present the *T_yx_* and *R_xx_* of our designed frequency-tunable reconfigurable multifunctional terahertz metasurface. It can be seen that when $\sigma_{{\mathrm{VO}}_{2},\mathrm{bottom}}$ = 2 × 10^5^ S/m and 2 × 10^2^ S/m, the resonant frequency was 1.59 THz and 1.73 THz, respectively. The transmissivity of cross-polarization and reflectivity of co-polarization were greater than 80% or less than 20% in the operating frequency.

**Figure 8:**
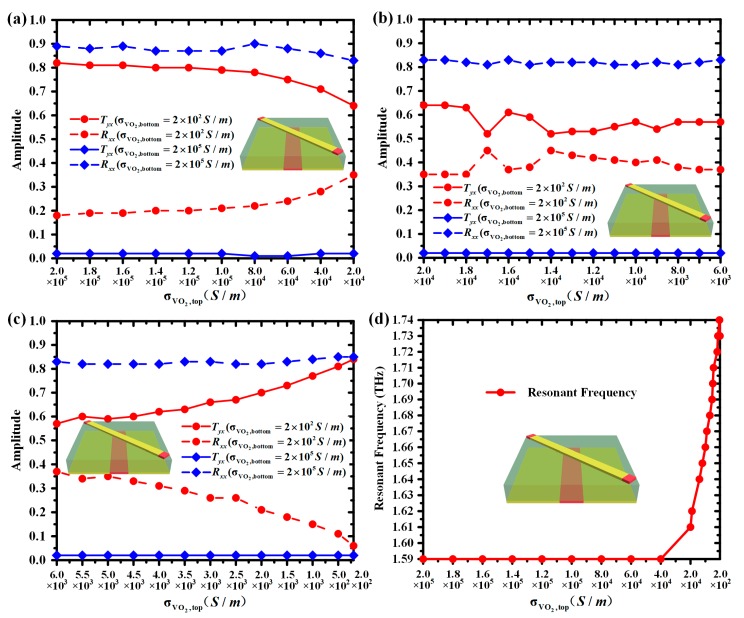
(**a**–**c**) When $\sigma_{{\mathrm{VO}}_{2},\mathrm{top}}$ ranges from 2 × 10^5^ S/m to 2 × 10^2^ S/m and $\sigma_{{\mathrm{VO}}_{2},\mathrm{bottom}}$ = 2 × 10^2^ S/m or 2 × 10^5^ S/m, the simulated amplitude of the cross-polarized transmission and co-polarized reflection coefficients (*T_yx_* and *R_xx_*) at the operating frequency; (**d**) When $\sigma_{{\mathrm{VO}}_{2},\mathrm{top}}$ varies from 2 × 10^5^ S/m to 2 × 10^2^ S/m, the different working frequency of the designed metasurface.

Figure 8 plots the *T_yx_*, *R_xx_* and working frequency for the different $\sigma_{{\mathrm{VO}}_{2},\mathrm{top}}$. It can be observed in Figure 8a–c that when $\sigma_{{\mathrm{VO}}_{2},\mathrm{top}}$ ranges from 2 × 10^5^ S/m to 2 × 10^2^ S/m with $\sigma_{{\mathrm{VO}}_{2},\mathrm{bottom}}$ = 2 × 10^2^ S/m, *T_yx_* changes between 0.52 and 0.82 while *R_xx_* varies between 0.11 and 0.45 and the mean value of *T_yx_* and *R_xx_* is about 0.65 and 0.31 at the working frequency, respectively. As $\sigma_{{\mathrm{VO}}_{2},\mathrm{top}}$ ranged from 2 × 10^5^ S/m to 2 × 10^2^ S/m with $\sigma_{{\mathrm{VO}}_{2},\mathrm{bottom}}$ = 2 × 10^5^ S/m at the working frequency, *T_yx_* and *R_xx_* remained essentially unchanged; the average value was about 0.11 and 0.81, respectively. In Figure 8d, the working frequency of the designed metasurface varies between 1.59 THz and 1.74 THz, while $\sigma_{{\mathrm{VO}}_{2},\mathrm{top}}$ decreased from 2 × 10^5^ S/m to 2 × 10^2^ S/m.

Therefore, it is clear that the working frequency can be effectively tuned between 1.59 THz and 1.74 THz by varying $\sigma_{{\mathrm{VO}}_{2},\mathrm{top}}$ without re-optimizing and re-fabricating the structures of the metasurface. Simultaneously, the high performance of diversified functionalities with transmission-reflection switching and polarization control can be maintained across 1.59–1.62 THz and 1.71–1.74 THz.

**Figure 9:**
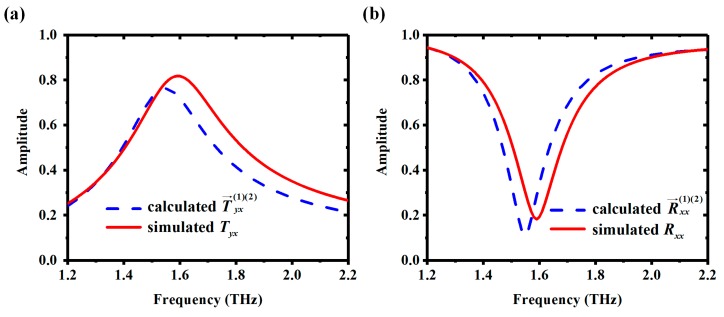
The analytical calculation of ${\vec{T}}_{yx}^{\left(1)(2\right)}$ and ${\vec{R}}_{xx}^{\left(1)(2\right)}$ (blue-dashed line) well approximates the *T_yx_* and *R_xx_* from the numerical simulation of the unit of the proposed metasurface (red-solid line). (**a**) The amplitude of ${\vec{T}}_{yx}^{\left(1)(2\right)}$ and *T_yx_*; (**b**) The amplitude of ${\vec{R}}_{xx}^{\left(1)(2\right)}$ and *R_xx_*.

Figure 9a,b show calculated ${\vec{T}}_{yx}^{\left(1)(2\right)}$ and ${\vec{R}}_{xx}^{\left(1)(2\right)}$ and simulated *T_yx_* and *R_xx_,* respectively, with $\sigma_{{\mathrm{VO}}_{2},\mathrm{bottom}}$ = 2 × 10^2^ S/m and $\sigma_{{\mathrm{VO}}_{2},\mathrm{top}}$ = 2 × 10^5^ S/m. According to Section 3, the overall ${\vec{T}}_{yx}^{\left(1)(2\right)}$ and ${\vec{R}}_{xx}^{\left(1)(2\right)}$ can be calculated by MATLAB based on flowcharts in Figure 5. Input scattering coefficients of two components in Figure 4b were obtained by simulation; the number of iterations *N* was 50. It can be observed that the calculated ${\vec{T}}_{yx}^{\left(1)(2\right)}$ and ${\vec{R}}_{xx}^{\left(1)(2\right)}$ agree well with the simulated *T_yx_* and *R_xx_*. The error between simulation and calculation results is due to the fact that the coupling between the top and bottom layer was not considered in the calculation of ${\vec{T}}_{yx}^{\left(1)(2\right)}$ and ${\vec{R}}_{xx}^{\left(1)(2\right)}$. Increasing the thickness *t* of the polyimide substrate will weaken the coupling effect between the top and bottom layer, which will also decrease the error.

Thus, it is clear that two diversified functions were integrated into the appropriately designed metasurface, and the working frequency can be dynamically tuned which effectively extend operating frequencies. The multifunctionality and operating frequency tunability can be realized by simply varying the conductivity of VO_2_ without re-optimizing or re-fabricating structures of the metasurface. The unit cell of the proposed metasurface consists of three simple layers. Compared with many complex unit cells of reconfigurable multifunctional metasurfaces, the proposed unit cell is maybe the simplest and most effective structure that can greatly reduce the manufacturing complexity and effectively acquire multifunctionalities. The proposed metasurface holds great potential for EM wave manipulation and can motivate the realization of the wideband multifunctional metasurface and the software-driven reconfigurable metasurface, which has the prospect to conveniently realize a complex system integration and device miniaturization with low costs at THz frequencies. This study can pave the way to many practical applications such as telecommunications, sensing and diagnostics, nanoelectronics, antennas and automotive.

## 5. Conclusions

In conclusion, we propose a reconfigurable multifunctional terahertz metasurface based on VO_2_. The designed metasurface can manipulate the linearized polarization state of EM waves and simultaneously realize the switch of transmission and reflection in the designed frequency range by utilizing the insulator-to-metal transition in VO_2_ inserted at the gap of the bottom metallic gratings; that is, this metasurface can convert incident waves into cross-polarized transmitted waves and co-polarized reflected waves in the designed frequency range. In addition, the operating frequency of this metasurface can be effectively tuned in the frequency range of 1.59 THz to 1.74 THz by varying the conductivity of the VO_2_ loaded on the top gold resonator from 2 × 10^5^ S/m to 2 × 10^2^ S/m, without re-optimizing and re-fabricating structures of the metasurface, which effectively extends the operating frequencies. The proposed metasurface holds great potential for EM wave manipulation and this study can motivate the realization of the wideband multifunctional metasurface and the software-driven reconfigurable metasurface at THz frequencies, which has the prospect to conveniently realize a complex system integration and device miniaturization with low costs.

The designed metasurface integrates only two expected functions; additionally, the performance still need to be optimized. Therefore, our future work may be focused on optimizing and experimentally verifying the performance of the designed metasurface. Photolithography could be used to fabricate the proposed metasurface, and the resistive heater or the external CW laser could be used to control the temperature of VO_2_. Moreover, an infrared camera could be employed to monitor the temperature. As an emerging research area, integrating multiple diversified functions into a single metasurface over a multiwavelength or wide wavelength range based on tunable metaparticles still requires dealing with formidable challenges at terahertz frequencies.
